# Antagonistic effect of ursolic acid on *Staphylococcal* biofilms

**DOI:** 10.14202/vetworld.2018.1440-1444

**Published:** 2018-10-17

**Authors:** J. Shiva Jyothi, Kalyani Putty, Y. Narasimha Reddy, K. Dhanalakshmi, M. A. Hannan Umair

**Affiliations:** Departments of Veterinary Microbiology, and Veterinary Biotechnology, College of Veterinary Science, P. V. Narsimha Rao Telangana Veterinary University, Rajendra Nagar, Hyderabad, Telangana, India

**Keywords:** biofilms, *icaD*, *Staphylococcus aureus*, ursolic acid

## Abstract

**Aim:**

The present study was carried out to study the effect of ursolic acid (UA) as a potential anti-biofilm agent in dispersing the biofilm generated by *Staphylococcus aureus* isolated from milk samples of crossbred dairy cows on the day of drying. Further, in the *S. aureus* isolates, the presence of intracellular adherence gene locus involved in biofilm production (*icaD*) was investigated.

**Materials and Methods:**

A total of 50 *S. aureus* strains were isolated over a period of 3 months from 200 milk samples collected from crossbred dairy cows on the day of drying. These isolates were subjected for biofilm detection by Congo red agar (CRA), microtiter plate assay (MTP), and polymerase chain reaction specific for *icaD* gene. The antagonistic effect of biofilm formation by UA was studied using different concentrations (30 µg/ml and 60 µg/ml) of UA and compared with the control group.

**Results:**

Among the 50 *S. aureus* subjected for biofilm detection, 34 and 40 isolates were detected as biofilm agents by CRA and MTP methods, respectively. The *in vitro* studies on the effect of UA in inhibiting biofilm formation by *S. aureus* using MTP assay showed 71.5% and 48.6% inhibition at UA concentrations of 60 µg/ml and 30 µg/ml, respectively, with a significant difference (p<0.05) between the treated and untreated isolates, which was further evident by scanning electron microscopy. Interestingly, the isolates that were tested to be resistant through Antibiotic Sensitivity Test to commonly used antibiotics were found to be sensitive to all the tested antibiotics following UA treatment at both the tested concentrations. Furthermore, molecular detection of *icaD* gene for biofilm detection revealed that all the isolates that were positive by MTP had *icaD* gene.

**Conclusion:**

Increased incidence of biofilm agents in dairy infections must be considered as an alarming situation. UA treatment significantly enhanced the sensitivity of the microbial pathogens to commonly used antibiotics. Hence, attention must be paid toward implementation of new strategies such as therapeutic regimes with a combination of antibiotic and anti-biofilm agents for effective treatment of infections in dairy farms.

## Introduction

Biofilm, also referred to as slime, is an extracellular polymeric conglomeration of DNA, proteins, and polysaccharides and is a significant problem in the medical, food, and marine industries often leading to substantial economic and health problems [1,2]. The microbial community of a biofilm is complex and highly resistant to antibiotics, posing a challenge of persistent infections despite antimicrobial therapies [3].

In the dairy industry, the ability of *Staphylococcus* species to produce biofilm is one of the primary reasons for treatment failure and recurrent infections of mammary gland [4]. Production of biofilm enables adhesion of bacteria to the epithelium of mammary glands and thus facilitates persistence in the host tissue and protection from host defense mechanisms. Production of biofilm mainly depends on the presence of the gene cluster *icaADBC* (the intracellular adhesion locus); strains harboring the *icaADBC* cluster are known to be potential biofilm producers [5]. Further, *icaA* and *icaD* were found to be in high prevalence among *Staphylococcus aureus* mastitis isolates implying that *ica* locus has a potential role as a virulence factor in the pathogenesis of mastitis in ruminants [6]. There are several conventional approaches to combating biofilms, physical and/or mechanical removal, chemical removal, and the use of antimicrobials, sanitizers, or disinfectants [4]. Recently, several compounds were tested for their efficacy in removal of biofilm generated by several microorganisms [7-19]. Ursolic acid (UA) (3-beta-3-hydroxy-urs-12-ene-28-oic-acid; UA) is a lipophilic pentacyclic triterpenoid and is widely found naturally in the peels of fruits, as well as in many herbs and spices such as lavender, oregano, thyme, rosemary, and thyme [20]. Previous studies suggested that UA from the tree *Diospyros dendo* added at the rate of 10 µg/ml decreased biofilm formation in *Escherichia coli, Vibrio harveyi*, and *Pseudomonas aeruginosa* by inducing chemotaxis and motility genes in bacteria [21].

The present study was carried out to study the effect of UA as a potential anti-biofilm agent in dispersing the biofilm generated by *S. aureus* isolated from milk samples of crossbred dairy cows on the day of drying. Further, in the *S. aureus* isolates, the presence of intracellular adherence gene locus involved in biofilm production (*icaD*) was investigated.

## Materials and Methods

### Ethical approval

Milk samples were collected with owners’ consent as per standard milk collection procedure. As no experimentation was performed on animals, ethical approval is not of concern to the present study.

### Sample collection

A total of 200 milk samples were collected aseptically over a period of 3 months from 200 crossbred dairy cows on the day of drying from various dairy farms in and around Hyderabad, Telangana.

### Bacterial isolation and phenotypic identification

Milk samples were centrifuged at 2000 g at 37°C for 10 min, supernatant was discarded, and 5 ml of brain heart infusion (BHI) broth was added to the sediment and incubated at 37°C for 24 h [22]. After incubation of milk samples in BHI broth, the morphology of the organisms was studied by Gram’s stain and cultural characters of the isolates were studied using mannitol salt agar. The isolates were also subjected to various standard biochemical tests [22,23].

### Biofilm detection by Congo red agar (CRA) and Microtiter plate assay (MTP) assay

Biofilm production was evaluated by the cultivation of *Staphylococcus* isolates on CRA plates [24]. Isolates were interpreted according to their colony phenotypes [6]. Quantification of biofilm formation by MTP was performed according to previous studies [25,26]. The optical density (OD) of each well was measured using a microplate ELISA reader BioTek (USA) at 630 nm. Cutoff OD (ODc) is defined as three standard deviations above the mean OD of the negative control. Strains were interpreted as follows: Non-biofilm producers (OD ≤ ODc); weak biofilm producers (ODc < OD ≤2× ODc); moderate biofilm producers (2× ODc < OD ≤4× ODc); and strong biofilm producers (4× ODc < OD).

### UA treatment

The antagonist effect of biofilm formation by UA was studied using different concentrations (30 µg/ml and 60 µg/ml) of UA and by its comparison with the control group that did not receive any treatment. For this, an overnight culture of biofilm-forming *S. aureus* was incubated for 12 h in tryptic soy broth (TSB) supplemented with 30 µg/ml and 60 µg/ml of UA (Sigma-Aldrich, CAS Number:7-55-1). Inhibitory effect was studied by MTP method as mentioned above. Anti-biofilm effect was detected by dividing the *S. aureus* isolates into three groups, control isolates (Group 1), UA-treated isolates at a concentration of 30 µg/ml (Group 2), and UA-treated isolates at the rate of 60 µg/ml (Group 3). The inhibitory rates were calculated using the formula:

Inhibitory rate (%) = OD (Control) - OD (Sample)/OD (Control) × 100%.

### Scanning electron microscopy

Scanning electron microscopic studies were conducted for three types of samples (a) non-biofilm *S. aureus*, (b) biofilm-forming *S. aureus*, and (c) *S. aureus* grown in the presence of the UA (30 µg/ml). Sample preparation was done as follows: (a) Non-biofilm *S. aureus*: Two to three pure isolated colonies were cut from the CRA plate and placed in glutaraldehyde. (b)` Biofilm-forming *S. aureus* and *S. aureus* grown in the presence of the UA: Colony of isolates positive for biofilm was inoculated into TSB and mixed thoroughly. 1 ml of the above was dispensed into each well of a 6-well tissue culture plate. Glass coverslip (0.2 mm thick and 6 mm in diameter) was immersed into the wells. Three wells were inoculated with 30 µg/ml of UA, and the wells without UA served as a control. The plate was incubated at 37°C for 18 h in shaker incubator, and the glass coverslips were used for the microscopic study. Samples were fixed in 2.5% glutaraldehyde in 0.1 M phosphate buffer (pH 7.2) for 24 h at 4°C followed by fixing in 2% aqueous osmium tetroxide for 4 h. These were then dehydrated in a series of graded alcohols. Processed samples were mounted over the stubs with double-sided carbon conductivity tape, and a thin layer of gold coat over the samples was done using an automated sputter coater (Model - Model JFC-1600) for 3 min. These were then scanned under scanning electron microscope (SEM - Model: JOEL-JSM5600) at required magnifications as per the standard procedures at RUSKA Labs, College of Veterinary Science, PVNRTVU, Rajendra Nagar, Hyderabad, India.

### Polymerase chain reaction (PCR)

Genomic DNA isolation of bacterial isolates was carried out by phenol-chloroform DNA extraction method. The extracted DNA was dissolved in 30 µl sterile distilled water and stored in −20°C and was used for the detection of biofilm formation gene (*icaD*) by PCR. Primers used in PCR to amplify *icaD* (*icaD*F:5’AAACGTAAGAGAGGTGG3’ and *ica*DR:5’ GGCAATATGATCAAGATAC 3’) were supplied by IDT™ Bangalore with an expected amplicon size of 381bp [6]. PCR reaction mixture was set up in 25 µl consisting of DNA template 2.5 µl, Taq buffer (10×) 2.5 µl, dNTPs (10 mM) 0.5 µl, forward primer (10 µM) 2.5 µl, reverse primer (10 µM) 2.5 µl, taq polymerase 1.0 µl, and nuclease-free water 13.5 µl.

### Antibiogram studies

Antibiogram was studied using antibiotic test discs manufactured by HiMedia Laboratories Limited, Mumbai and Oxoid, UK, with principle of disc diffusion method of Kirby and Bauer [22]. The antibiotics and their concentration used in the present study were ampicillin (10 µg), ceftiofur (30 µg), enrofloxacin (30 µg), gentamicin (30 µg), methicillin (30 µg), and tetracycline (30 µg). The interpretations of test were carried out according to CLSI guidelines (2013).

### Statistical analysis

Data were collected and analyzed using Excel and SPSS Statistics for Windows, Version 23.0 (IBM, USA). One-way ANOVA was carried for comparing the effect of UA in inhibiting biofilm formation; p<0.05 was considered as statistically significant for univariate analysis.

## Results

### Bacterial isolates and identification

The isolated bacteria from dairy cows were identified by conventional methods. All of the 50 strains were found Gram-positive and catalase-positive cocci. All the 50 strains were rabbit plasma coagulase positive and were considered as *S. aureus*.

### Biofilm detection

**Figure-1:**
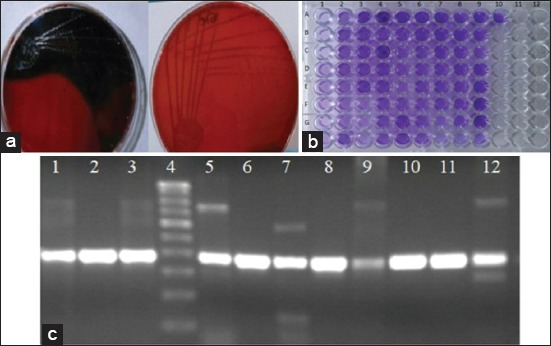
Biofilm evaluation methods of *Staphylococcus aureus*. (a) Differentiation of biofilm producers and non-biofilm producers on Congo red agar: Biofilm-forming *S. aureus* isolates produce black colonics on Congo red agar (left), as opposed to non-biofilm forming S. aureus, isolates that produce red colonies on Congo red agar (right). (b) Phenotypic characterization of biofilm formation by *S. aureus* isolates in microtiter plate test: Strong ability to form biofilms can be seen in wells with darker shade of blue (e.g., G9), moderate ability can be seen in wells with medium shade of blue (e.g., H5), and weak ability can be seen in wells with light shade of blue (e.g., H7). (c) (idiotypic characterization of biofilm-producing *S. aureus*: icaD gene was amplified from all the tested isolates (381 bp: Lane 1, 2, 3, 5, 6, 7, 8, 9, 10, 11, and 12) Lane4: 100 bp DNA Ladder.

Among the 50 *S. aureus* subjected for biofilm detection, 34 and 40 isolates were detected as biofilm agents by CRA and MTP methods, respectively. The 40 isolates tested to be as biofilm producers were further tested for the presence of *icaD* gene and all were found to be positive for the presence of the gene (Figure-1).

### Study of inhibitory rates (%) for biofilm formation by UA

**Figure-2:**
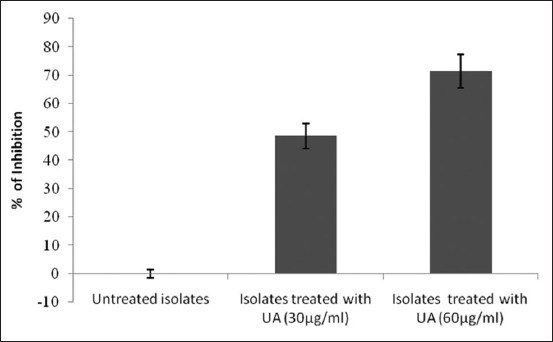
Inhibition of biofilm formation of isolates treated with U A. As tested by MTP assay, *Staphylococcus aureus* isolates treated with UA at concentrations of 30 pg/ml and 60 pg/ml showed an inhibitory rate of 48.6% and 71.5%, respectively, when compared with untreated isolates.

Biofilm-forming *S. aureus* isolates treated with UA (30 µg/ml) showed an inhibitory rate of 48.6% when compared to that of the isolates that were untreated, as tested by MTP assay. Further, the inhibitory percentage of biofilm formation of the *S. aureus* isolates when treated with UA at a concentration of 60 µg/ml had increased to 71.5% (p<0.05) (Figure-2).

### Scanning electron microscopy

**Figure-3:**
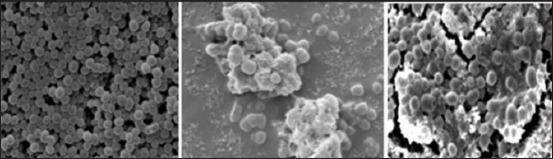
Dissolution of biofilm following UA treatment. It scanning electron microscopic image of non-biofilm *Staphylococcus aureus* (left). Biofilm-forming *Staphylococcus aureus* with intracellular adhesions and thick matrix (middle), and biofilm-forming *S. aureus* treated with UA showing decreased intracellular adhesions and thin matrix layer (right).

Electron microscopic studies showed that non-biofilm *S. aureus* was in bunches with no extracellular matrix layer around the isolates. For biofilm-producing *S. aureus*, intracellular adhesions with a thick extracellular matrix layer were clearly seen. For isolates grown in the presence of UA, a decrease in the matrix layer was observed (Figure-3).

### Antibiogram studies

**Figure-4:**
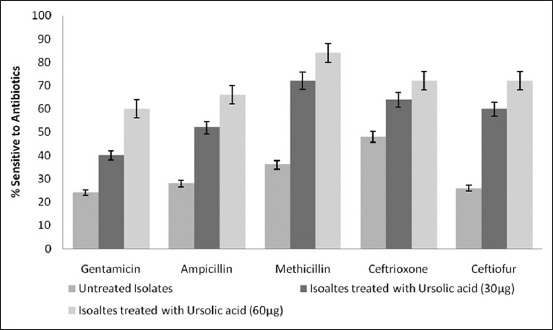
Increased sensitivity of *S. aureus* isolates to antibiotics following UA treatment. Increase in sensitivity to different antibiotics was noticed on the treatment of the *S. aureus* isolates with different concentrations of UA.

The antibiogram pattern of *S. aureus* isolates treated with UA (30 µg/ml and 60 µg/ml) was compared with the untreated isolates. The results showed that hitherto-resistant isolates to various antibiotics showed increased sensitivity to the same antibiotics when treated with UA as compared to untreated isolates (Figure-4).

## Discussion

*S. aureus* is a major etiological agent of bovine mastitis [27]. The losses due to mastitis are not only economical, but the side effects that ensue mastitis treatment strategies are even more alarming; major one being antimicrobial resistance due to indiscriminate use of antibiotics. Moreover, biofilm formation by certain bacterial species is known to be triggered by indiscriminate antibiotic usage [28]. Adding to it, reports of alarming increase in cases of methicillin-resistant *S. aureus* (MRSA) warrants immediate attention towards the control of resistant strains of *S. aureus* in clinical settings. Our findings revealed biofilm formation ability of mastitis pathogens, especially *S. aureus* isolated from milk samples on the day of drying. This suggests that efficient dry cow therapy with optimal dosage of antibiotics needs interventions in the form of anti-biofilm agents for efficient elimination and prevention of infections.

In the current study, detection of *icaD* gene in biofilm-forming *S. aureus* isolates was in accordance with findings of Samah *et al*. [29], who detected *icaD* gene in 62.5% of coagulase-positive *S. aureus* and 47.1% in coagulase-negative *S. aureus* isolated from mastitis. Considering the nature of biofilms in preventing the action of the antibiotics, we further tested the ability of UA as an anti-biofilm agent to disperse the biofilm layer around the organism and in doing so, enhanced sensitivity of the strains to the same antibiotics was also determined. UA treatment was known to inhibit biofilm formation by MRSA [30]. In the current study, UA was found to significantly disperse the biofilm around the microorganisms which could explain the enhanced sensitivity of these strains to the antibiotics which the isolates were resistant to before UA treatment. Further, to look into if there exists any relation between the biofilm production, methicillin resistance, and leukocidin toxicity; we have subjected all the 40 positive *icaD* gene isolates for the detection of *mec*A and *pvl* gene. Interestingly, 15 isolates of the 40 positive *icaD* gene isolates have shown the presence of *mec*A gene, and none of the isolates were positive for *pvl* gene (data not shown). The absence of *pvl* gene in the tested isolates of *S. aureus* indicated the absence of leukocidin toxin which could be due to the fact that the isolates in the current study were from subclinical cases and not from the clinical cases [31]. The prevalence of MRSA in the *S. aureus* isolates of the current study emphasizes the importance of failure in treatment strategies of bovine mastitis. Treatment strategies aimed toward a prophylactic approach of combining antimicrobials with biofilm dispersal agents can aid in combating the biofilms, thereby making the pathogenic microorganisms susceptible to antimicrobials in an efficient way [32-34].

## Conclusion

Findings of the present study demonstrated a great ability of *S. aureus* isolates to form biofilms, even in the subclinical cases. The presence of such bacteria in the microenvironment of the udder may lead to the inefficacy of antibiotic treatment of dry cow therapy or clinical mastitis and warrants an urgent need for understanding the biofilm-forming capabilities of mastitis pathogens in designing better treatment strategies. The ability of UA as a potential antagonist of biofilms could be useful in adjunct therapies for the treatment of biofilm-involved infections and initiates the need for understanding the ability of more such compounds.

## Authors’ Contributions

JSJ, KP, YNR, and KD designed the study. JSJ, KP, and MAHU performed the experiments. JSJ and KP analyzed the data and wrote the manuscript. All authors read and approved the manuscript.
